# Preparation of Maleic Anhydride Grafted Polybutene and Its Application in Isotactic Polybutene-1/Microcrystalline Cellulose Composites

**DOI:** 10.3390/polym10040393

**Published:** 2018-04-02

**Authors:** Bo Wang, Kai Nie, Xiao-rong Xue, Fu-hua Lin, Xiang-yang Li, Yong-bing Xue, Jun Luo

**Affiliations:** 1 School of Chemical and Biological Technology, Taiyuan University of Science and Technology, Taiyuan 030021, China; 13546474299@163.com (B.W.); 18235186883@163.com (K.N.); r1044381617@163.com (X.-r.X.); 2 Key Laboratory of Renewable Energy, Guangzhou Institute of Energy Conversion, Chinese Academy of Sciences, Guangzhou 510640, China; kelin0514@163.com; 3 Shanxi Provincial Institute of Chemical Industry, Taiyuan 030021, China; 17835611229@163.com; 4 Guangzhou Fibre Product Testing and Research Institute, Guangzhou 510220, China

**Keywords:** maleic anhydride grafted polybutene, isotactic polybutene-1, microcrystalline cellulose, composite, compatibilizer

## Abstract

Microcrystalline cellulose (MCC) offers great potential to improve the mechanical and crystallization properties of isotactic polybutene-1 (iPB) because of its low cost, biodegradability, renewability and excellent mechanical properties. However, the compatibility of polar MCC and non-polar iPB is poor. In this study, maleic anhydride grafted polybutene (MAPB) was prepared by the solution method and was used as a compatibilizer in the fabrication of iPB/MCC composites by using a twin screw extruder. The ultimate tensile strength, tensile modulus, flexural strength, flexural modulus of the iPB/MCC composites increased by 3.1%, 16.5%, 10.7%, 6.5%, respectively, compared with that of pure iPB. With MAPB addition, these values increased by 17.2%, 31%, 17.5% and 10%, respectively, compared with that of pure iPB. The heat-distortion temperature and thermal-decomposition temperature of all composites increased with an increased MCC content. The non-isothermal crystallization of the iPB/MCC composites shows that MCC addition can promote iPB crystallization, because the non-isothermal crystallization curve of the composites moves toward a higher temperature, especially after MAPB addition. Scanning electron micrographs indicate that the compatibility of the iPB/MCC has been enhanced significantly.

## 1. Introduction

Isotactic polybutene-1 (iPB) is a semi-crystalline polymer, which has been used extensively in hot-water systems, heating systems, wires, cables and other industries. iPB has many excellent properties, such as an excellent heat, creep, pressure resistance [1,2]. However, its high cost and slow crystallization rate limit its extensive use. Because iPB is a type of polymorphic form polymer, initially, it is generated as form II after heat processing and then it changes into form I over a period of as long as 7 days [3,4]. To expand the scope of application of iPB, blending and chemical modifications have been proposed [5,6]. Among them, blending modification has advantages of a convenient operation and an economic efficiency [7]. The current filler that is used for iPB blending modification contains the inorganic and organic polymer filler. Iervolino et al. [8] studied the crystallization behavior of iPB/multi-walled carbon nanotube (MWNT) composites. The nucleating effect on the iPB is attributed to the MWNT addition. However, the high cost of MWNTs restricts its wide use. Li et al. [9] used extrusion and injection to prepare an iPB/polypropylene (PP) composite and studied its mechanical properties. The tensile strength and elongation at break of the blends decreased, but the impact and flexural properties were improved by PP addition. This technology resolves the problem of the iPB expense, but the crystallization properties of the iPB remain unchanged. Therefore, the sourcing of a cheap and biodegradable fiber could theoretically reduce the price and improve the iPB performance.

Microcrystalline cellulose (MCC) is a type of biodegradable polymer fiber that is used extensively in medicine, food and other fields [10,11,12]. In recent years, MCC has offered great potential as a type of filler to prepare polymer composites because of its low cost, low density, biodegradability, renewability [13,14,15]. In recent years, MCC use as a filler to prepare composites of excellent performance has attracted increased attention. Mathew et al. [16] prepared a polylactic acid (PLA)/MCC composite; the composite crystallinity was improved significantly with MCC addition, which can cause an increase in the tensile modulus of the composite. The MCC was applied to engineering thermoplastic composite nylon-6 by Kiziltas et al. [17] to yield a composite with improved mechanical properties.

However, iPB and MCC are usually incompatible because of the non-polarity of the iPB and the polar character of the MCC, which lead to a weak iPB/MCC interfacial adhesion and limits the composite application. A compatibilizer is used to improve the compatibility of the composite interfaces. Additives are used in most composites because of their convenient operation and good modification effect [18]. Of the compatibilizers, maleic anhydride graft copolymer improves the interfacial adhesion between the cellulosic filler and polyolefin matrix significantly [19]. The maleic anhydride graft copolymer molecular chain contains carboxyl and polymer molecular chains that can form hydrogen bonds with hydroxyl groups on the MCC surface, co-crystallize with the polymer matrix, thus play the role of compatibilizer. Therefore, maleic anhydride grafted polybutene (MAPB) as a compatibilizer can be used to improve the compatibility of iPB and MCC and could yield good results.

In this study, MAPB was prepared by the solution method and its structure and properties were characterized by Fourier-transform infrared spectroscopy (FTIR) and elemental analysis. iPB/MCC composites were prepared by melt blending. The effects of MCC and MAPB addition on the mechanical, thermal, crystallization properties and composite microstructural changes were investigated.

## 2. Materials and Methods

### 2.1. Materials

iPB (P5050) with a melt flow index of 200 g/10 min was supplied by Mitsui Chemical Inc. Dicumyl peroxide (DCP) was from Shanghai Weifang Fine Chemical Co., Ltd. (Shanghai, China). KOH, acetone, xylene, ethanol, HCl were from Tianjin Tianli Chemical Reagents Co., Ltd. (Tianjin, China) Maleic anhydride (MAH) was from Tianjin Damao Chemical Reagent Factory (Tianjin, China).

### 2.2. MAPB Preparation

iPB (5 g) and MAH (0.25 g) were dissolved in xylene (100 mL) in a three-necked flask that was heated rapidly to 125 °C. DCP (0.03 g) was added and the temperature was maintained at 125 °C for 5 h. The clear solution was poured into cold acetone (250 mL, 0 °C) to form a precipitate. The product was purified using acetone at 80 °C for 12 h by Soxhlet extraction and followed by filtration to remove the unreacted MAH and to obtain solid MAPB product (grafting rate (G) = 0.42%). G was obtained by the titration method [20].

MAPB (0.5 g) was dissolved in xylene at 125 °C for 1 h. KOH–ethanol solution (5 mL, 0.1 mol/L) was added into the system for 15 min. Then, two drops of phenolphthalein were added into the solution and the solution changed to pink. Finally, the pink solution was titrated using HCl–ethanol solution (0.1 mol/L) until the solution was colorless, G was calculated from:

(1) $$G=\frac{{\;(N}_{1}V_{1}-N_{2}V_{2})\times 98{.06\times 10}^{-3}}{2m}$$

where N_1_ (mol/L) is the concentration of the KOH–ethanol solution and V_1_ (mL) is the volume of the KOH-ethanol solution. N_2_ (mol/L) is the concentration of the HCl–ethanol solution, V_2_ (mL) is the volume of the HCl-ethanol solution, m (g) is the mass of MAPB.

### 2.3. MAPB Characterization

#### 2.3.1. FTIR Analysis

In preparation for FTIR, samples were pressed into slices by a heating pressing machine (120 °C, 12 MPa) for 3 min. FTIR was performed on a FTIR spectrometer (Nicolet iS50, Thermo Scientific Inc., Waltham, MA, USA) using 64 scans per sample.

#### 2.3.2. Elemental Analysis

Percentages of elemental C, O, H in each sample were determined by using an organic element analyzer (Vario EL Cube, Elementar Inc., Langenselbold, Germany). The test temperature was 1000 °C and the helium flow rate was 90 mL/min.

### 2.4. Preparation of iPB/MCC Composites

**Figure 1:**
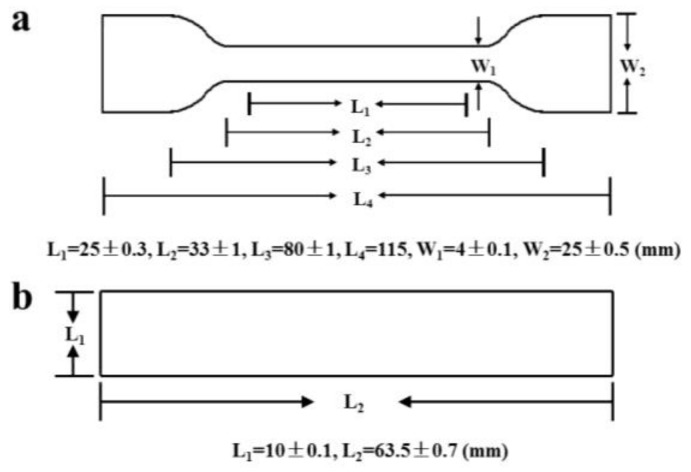
Standard test specimen size. (**a**) for tensile properties test; (**b**) for impact strength, flexural properties and heat-distortion temperature test.

The iPB/MCC composites (see composite formulae and sample codes in Table 1) were prepared by using a twin screw extruder (Nanjing Jieya Extrusion Equipment CO., Ltd. SHJ-20, Nanjing, China). The extruder temperature of each division was 110, 140, 160, 166, 170 °C. Standard test specimens (Figure 1) of the iPB/MCC composites were molded by an injection machine (Fomtec Machinery Co., Ltd., FT-150, Qingdao, China) with an injection pressure of 10 MPa at 140 °C.

### 2.5. Characterization of iPB/MCC Composites

#### 2.5.1. Mechanical Properties

Composite tensile properties and flexural properties were evaluated by using a universal testing machine (Z020, Zwick Roell Group, Ulm, Germany) at a crosshead speed of 5 mm/min at room temperature according to GB/T 1040-2006 and GB/T 9341-2008. The calculation of tensile modulus (E) is as follow formula:

(2) $$E=\frac{\sigma_{2}{-\sigma }_{1}}{\varepsilon_{2}{-\varepsilon }_{1}}$$

where σ is the tensile stress, ε is the tensile strain, ε_2_ = 0.0025, ε_1_ = 0.0005, σ_2_ and σ_1_ is the tensile stress corresponding to ε_2_ and ε_1_.

The impact strength of the composites was measured by an impact tester (Ceast 9050, Instron Company, Norwood, MA, USA) with an 11-J capacity at a maximum pendulum height (150°) at room temperature according to GB/T 1043.1-2008.

For the mechanical-properties test, each iPB/MCC composite was test by using 5 samples and the average results were recorded.

#### 2.5.2. Heat-Distortion Temperature

Heat-distortion-temperature (HDT) tests were performed by using HDT/VICAT equipment (CEAST HV500, Instron Company, Norwood, MA, USA) at a heating rate of 2 °C/min and a load of 1.82 MPa according to GB/T 1634.2-2004. Each iPB/MCC composite was tested by using 10 samples and the average results were recorded.

#### 2.5.3. Scanning Electron Microscopy

Samples iPB/MCC5 and M-iPB/MCC5 were observed via scanning electron microscopy (SEM) (S-4800, Hitachi, Tokyo, Japan) at an accelerating voltage of 10 kV to observe the compatibility at the fracture surface of the composites. An image analysis program (ImageJ) was used to measure the particle size distribution of the samples from the micrographs. More than 100 particles in each sample were examined.

#### 2.5.4. Thermogravimetric Analysis

Thermogravimetric (TG) analysis was conducted by using a thermogravimetric analyzer (Q500, TA Instrument Inc., New Castle, DE, USA) under nitrogen. The thermal stability of the samples (10 ± 1 mg) was scanned from 100 °C to 550 °C at 10 °C/min.

#### 2.5.5. Differential Scanning Calorimetry Analysis

Differential scanning calorimetry (DSC) analysis was carried out on a differential scanning calorimeter (Q2000, TA Instrument Inc., New Castle, DE, USA). The samples (10 ± 1 mg) were heated to 200 °C at 10 °C/min, maintained at 200 °C for 5 min to erase the thermal history cooled to 50 °C at 10 °C/min. Next, samples were heated to 200 °C at 10 °C/min. The heat flows during crystallization and melting were recorded for subsequent data analysis.

## 3. Results and Discussion

### 3.1. FTIR Spectra of iPB and MAPB

**Figure 2:**
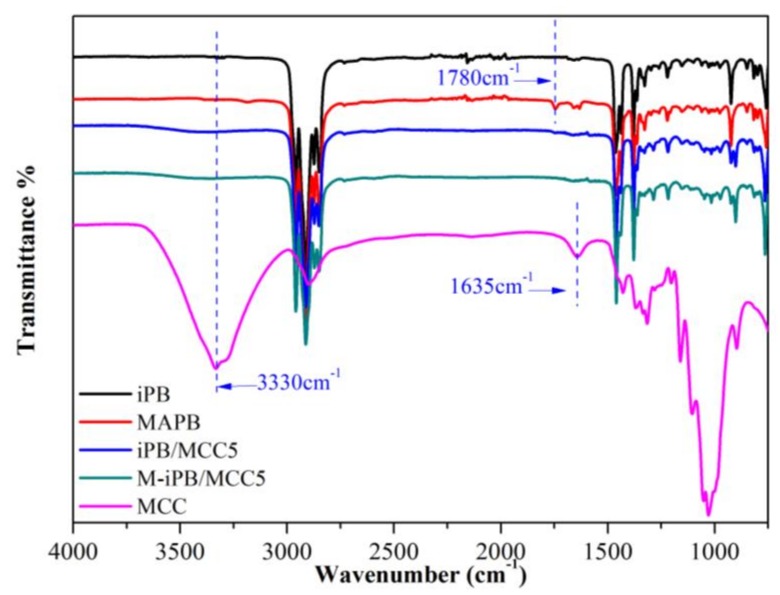
**Figure** **2** FTIR spectrum of the samples.

The FTIR spectra of the samples are shown in Figure 2. The stretching vibration peak of –CH_3_ in the iPB molecular chain appears at 2973 cm^−1^, 1467 cm^−1^, 1379 cm^−1^. The vibration of –CH_2_ appears at 2900 cm^−1^ and 2849 cm^−1^. In addition, the spectrum of the MCC showed a characteristic peak near 3330 cm^−1^, which is related to the hydroxyl stretching vibration of cellulose and the peak at 1635 cm^−1^ (the hydroxyl stretching vibration of adsorbed water). A characteristic peak of the hydroxyl group appeared in the FTIR spectrum of the iPB/MCC composites. A new peak at 1780 cm^−1^ in the FTIR spectrum of MAPB corresponds to the C=O stretching vibration, which proves that MAH has been grafted with iPB [21].

### 3.2. Elemental Analysis of iPB and MAPB

The elemental analyses of the iPB and MAPB are shown in Table 2. Compared with iPB, the elemental C in the MAPB decreased to 85.28% from 85.89%, the elemental H increased to 14.24% from 14.11%, the elemental O increased to 0.38% from 0%. This proves that elements in the samples contained O which indicates that MAH was grafted onto the molecular chain of the iPB.

### 3.3. Mechanical Properties of iPB/MCC Composites

**Figure 3:**
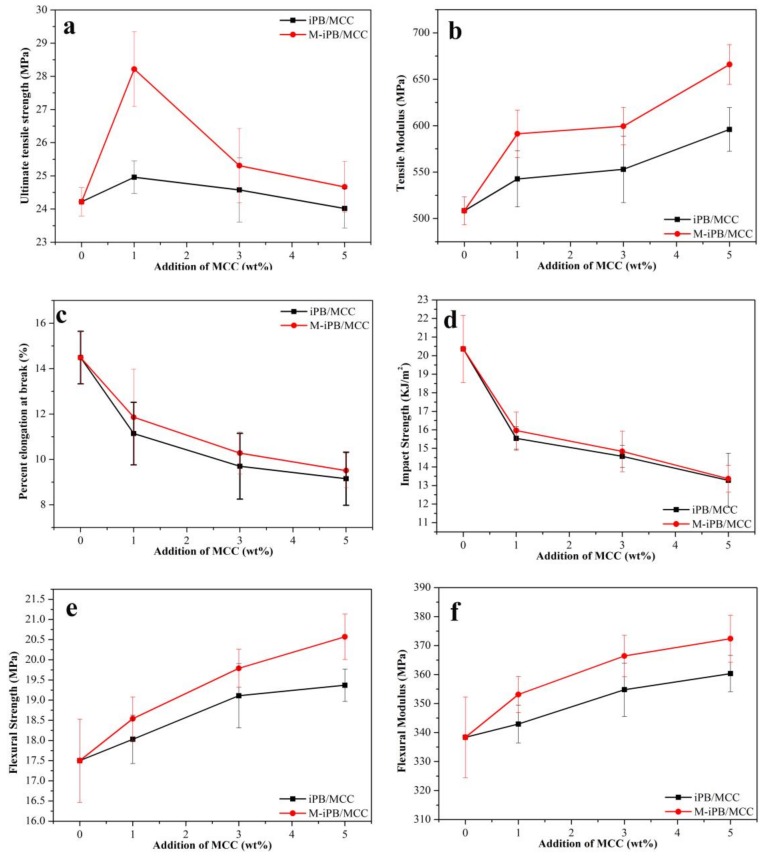
Mechanical properties of iPB/MCC composites (**a**) ultimate tensile strength; (**b**) tensile modulus; (**c**) percent elongation at break; (**d**) impact strength; (**e**) flexural strength; (**f**) flexural modulus).

The mechanical properties of iPB/MCC composites were shown in Figure 3. With MCC addition, the ultimate tensile strength (Figure 3a) of the composite first increased and then decreased. The maximum values (1% MCC added) increased from 24.22 MPa to 24.96 MPa (increase of 3.1%) for the iPB/MCC and to 28.22 MPa (increase of 16.5%) for the M-iPB/MCC. The MCC may have influenced the tensile properties of the composites, but hindered the plasticizing effect with a further increase in MCC content [22]. The MAPB addition increased the ultimate tensile strength of the M-iPB/MCC composites significantly. This occurred because the MAPB branches reacted with the hydroxyl groups of the MCC to provide a stress transfer between the iPB and MCC, which improved the compatibility of the iPB and MCC [23]. The tensile modulus (Figure 3b) of the composites increased from 508.35 MPa to 595.97 MPa (5% MCC added, increase of 17.2%) and to 665.93 MPa (5% MCC and 2% MAPB added, increase of 31%) compared with pure iPB. The main reason for this result may be that the MAPB may change the molecular morphology of the polymer chains near the iPB-MCC interphase improve the compatibility of the iPB and MCC [24]. The percent elongation at break (Figure 3c) of the composites decreased significantly with an increase in MCC content. The main reason may be that MCC has a lower percent elongation at break and may restrict the flow of polymer molecules past one another [25]. This behavior has been reported by many researchers [26,27,28]. The impact strength of the composites (Figure 3d) decreased from 20.36 kJ/m^2^ to 13.28 kJ/m^2^ for the iPB/MCC and to 13.36 kJ/m^2^ for the M-iPB/MCC with an increase in MCC content. The main reason for this result may be that the MCC was less flexible than the iPB, which makes the movement of iPB molecular chains more difficult with MCC addition. Figure 3e shows the flexural strength of the iPB/MCC composites. With an increase in MCC content, the flexural strength of all the composites increased significantly from 17.5 MPa to 19.37 MPa (5% MCC added, increase of 10.7%) for the iPB/MCC and 20.57 MPa (5% MCC added, increase of 17.5%) for the M-iPB/MCC. This result indicates that the compatibility between the iPB and MCC improved [29]. Figure 3f shows the flexural modulus of the iPB/MCC composites. The flexural modulus of the M-iPB/MCC composites was higher than that of the iPB/MCC composites. The modulus also increased with an increased MCC loading and reached 360.35 MPa (5% MCC added, increase of 6.5%) and 372.36 MPa (5% MCC added, increase of 10%) with the addition of 5 wt% MCC. This result is attributed to the flexural modulus being very sensitive to the matrix properties and fiber matrix interfacial bonding and the improved adhesion between the iPB and the MCC [30].

### 3.4. HDT of iPB/MCC Composites

**Figure 4:**
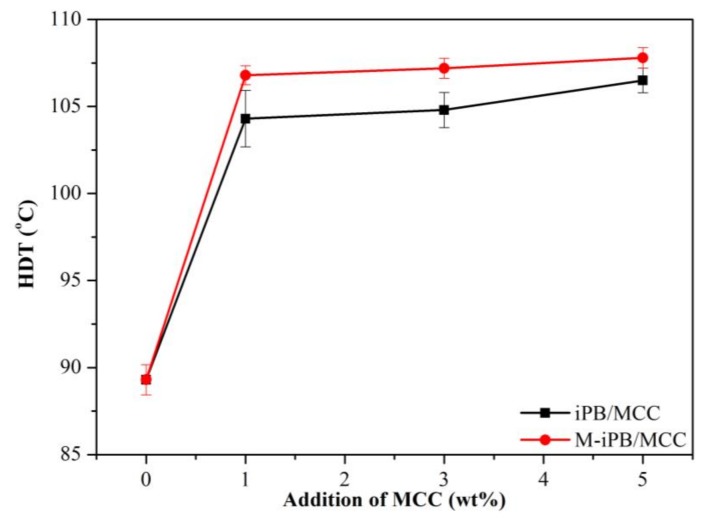
Heat-distortion-temperature (HDT) of iPB/MCC composites.

The HDT of different samples is reported in Figure 4. The HDT of the iPB was 89.3 °C. The HDT of the iPB/MCC composites increased with an increase in MCC concentration reached 106.5 °C and 107.8 °C. This result indicates that the thermal stability of the composite improved because MCC addition hindered the movement of iPB chains. The HDT of the M-iPB/MCC composites with the same ratio tended to be higher than that of the iPB/MCC composites. This means that the M-iPB/MCC has a better thermal stability than the iPB/MCC and the MAPB improved the compatibility of the iPB and MCC.

### 3.5. SEM Micrograph of iPB/MCC Composites

**Figure 5:**
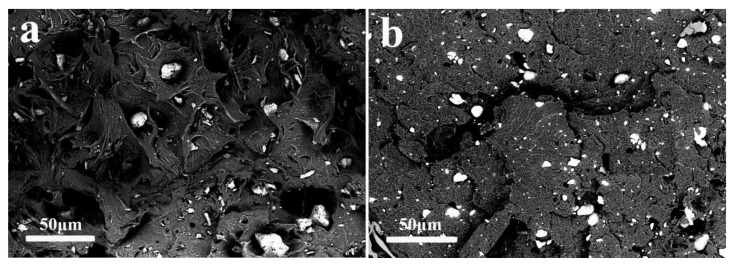
SEM micrograph of iPB/MCC composites (**a**) iPB/MCC5; (**b**) M-iPB/MCC5.

**Figure 6:**
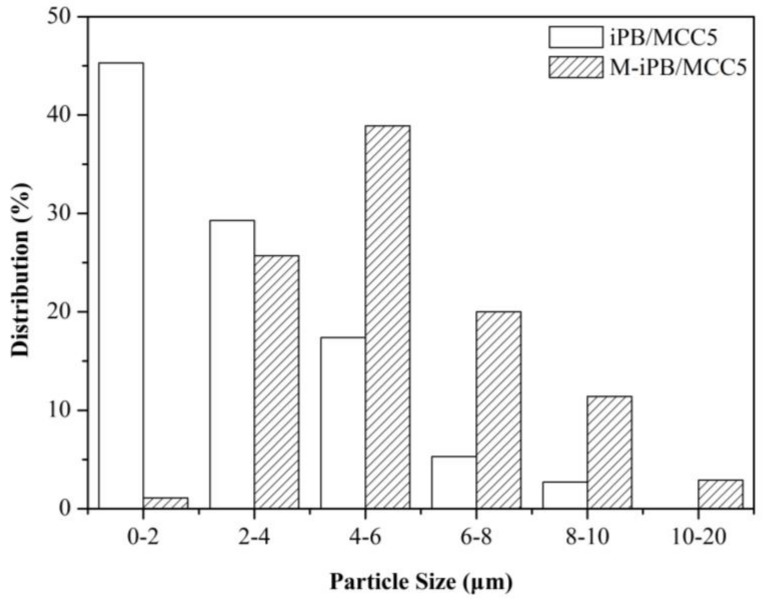
Particle size distribution of iPB/MCC composites.

To study the compatibility of the iPB and MCC, a SEM micrograph of the iPB/MCC composites is shown in Figure 5. The microstructure of the iPB/MCC composite is divided into an iPB phase (black) and the MCC phase (white). The particle size and pores of the MCC that remain in the iPB/MCC are relatively large the particle size is smaller fewer MCC pores remain in the M-iPB/MCC5. The particle size distribution of MCC in the iPB matrix (Figure 6) shows that the particle size of MCC in the iPB matrix decreased significantly with MAPB addition and the dispersion improved greatly. This result proves that the compatibility of MCC and iPB improves. The interfacial adhesion ability and dispersion property increased with MAPB addition, which shows a good compatibility. The properties of the iPB/MCC composites increased with the enhanced compatibility.

### 3.6. DTG of iPB/MCC Composites

**Figure 7:**
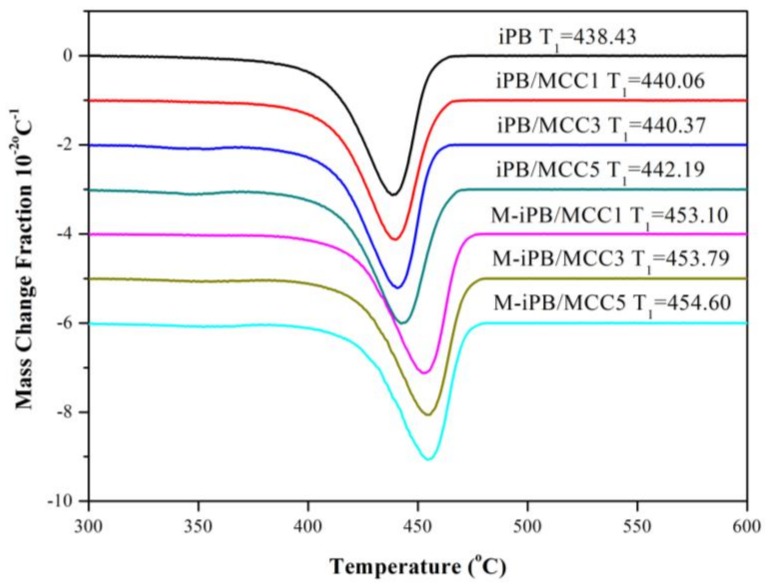
Derivative thermogravimetric analysis (DTG) curve of iPB/MCC composites.

The DTG curves of the iPB/MCC composites are shown in Figure 7. T_1_ is the temperature of the maximum complete mass loss in a single degradation step for the iPB/MCC composites. The single degradation steps between 370 and 500 °C correspond to the thermal decomposition of the polymer. With an increase in MCC loading in the iPB/MCC composites, the T_1_ values shift to a higher temperature, which indicates that the MCC affects the thermal-degradation process. Compared with the iPB/MCC composites, the addition of MAPB increased the T_1_ significantly. The thermal stability of the M-iPB/MCC composites was higher than that of the iPB/MCC composites. The above conclusions are consistent with the HDT analysis, which indicated that MCC addition contributes to an improvement in thermal properties of the composites with an improvement in the compatibility of the iPB and MCC, the thermal properties of the composites increase.

### 3.7. Non-Isothermal Crystallization of iPB/MCC Composites

**Figure 8:**
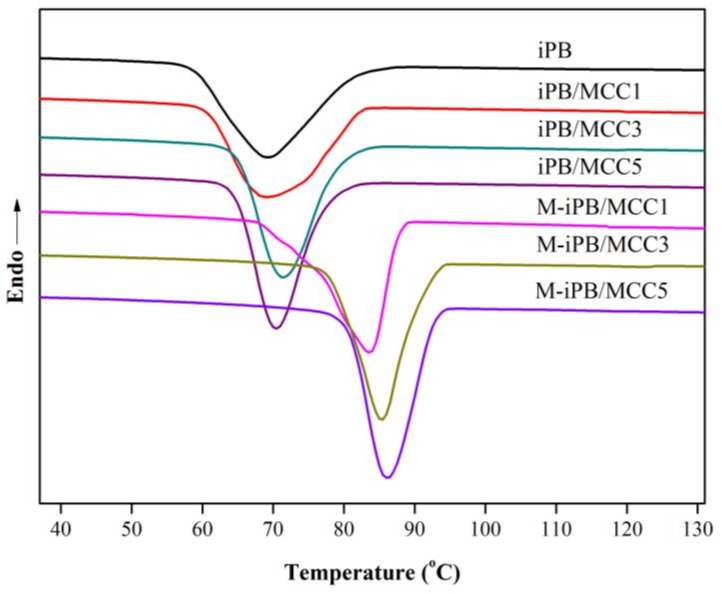
Non-isothermal crystallization DSC curve of iPB/MCC composites.

The non-isothermal crystallization DSC curve of the iPB/MCC composites is shown in Figure 8. Compared with iPB, the peak crystallization temperature of the iPB/MCC composites increased, which indicates that the addition of MCC promoted the crystallization ability of iPB. The crystallization peak width narrowed, which indicates that the addition of MCC shortens the crystallization time and increases the crystallization rate of the iPB. Compared with the iPB/MCC composites, the addition of MAPB increased the peak crystallization temperature of the M-iPB/MCC significantly more than that of the iPB/MCC, which proves that the iPB and MCC compatibility and MCC nucleation improve.

## 4. Conclusions

MAPB was prepared by the solution method. The FTIR and elemental analysis results indicate that the MAH group was indeed grafted onto the iPB molecular chain. The iPB/MCC composite was prepared by melt blending. MCC addition compared with pure iPB resulted in an increase in ultimate tensile strength, tensile modulus, flexural strength and flexural modulus of the iPB/MCC composites by 3.1%, 16.5%, 10.7%, 6.5%, respectively. With MAPB addition, these values increased by 17.2%, 31%, 17.5%, 10%, respectively. These results indicate that the MAPB can improve the compatibility of iPB and MCC significantly. However, the impact strength and percent elongation at break of all composites decreased with a higher MCC content. The HDT of the iPB/MCC composites increased with a higher MCC content and the HDT of the composites with MAPB was higher than that without MAPB. A similar behavior was observed from the DTG analysis and non-isothermal crystallization of the composites. The entire phenomenon proves that the MAPB improves the interfacial compatibility of the iPB/MCC composites, which leads to an increase in the mechanical, thermal and crystallization properties. SEM micrographs and the particle size distribution of the iPB/MCC show that the compatibility has been enhanced significantly with MAPB addition.

## Figures and Tables

**Table 1:** Formulae of the Isotactic Polybutene-1(iPB)/Microcrystalline cellulose (MCC) composites.

Sample	iPB (g)	MCC (g)	MAPB (g)
iPB	1000	-	-
iPB/MCC1	1000	10	-
iPB/MCC3	1000	30	-
iPB/MCC5	1000	50	-
M-iPB/MCC1	1000	10	20
M-iPB/MCC3	1000	30	20
M-iPB/MCC5	1000	50	20

**Table 2:** Elemental analysis of iPB and maleic anhydride grafted polybutene (MAPB).

Sample	C (%)	H (%)	O (%)
iPB	85.89	14.11	0
MAPB	85.28	14.24	0.38
